# Applications of Functional Amyloids from Fungi: Surface Modification by Class I Hydrophobins

**DOI:** 10.3390/biom7030045

**Published:** 2017-06-26

**Authors:** Alessandra Piscitelli, Paola Cicatiello, Alfredo Maria Gravagnuolo, Ilaria Sorrentino, Cinzia Pezzella, Paola Giardina

**Affiliations:** 1 Department of Chemical Sciences, Università degli Studi di Napoli Federico II, Complesso Universitario Monte S. Angelo, Via Cintia 4, 80126 Naples, Italy; apiscite@unina.it (A.P.); paola.cicatiello@unina.it (P.C.); alfredo.gravagnuolo@manchester.ac.uk (A.M.G.); ilaria.sorrentino@unina.it (I.S.); cpezzella@unina.it (C.P.); 2 Division of Pharmacy and Optometry, Faculty of Biology, Medicine and Health, The University of Manchester, M13 9PT Manchester, UK

**Keywords:** functionalization, adhesion, biosensors, protein immobilization, biomedical applications, nanomaterials

## Abstract

Class I hydrophobins produced from fungi are amongst the first proteins recognized as functional amyloids. They are amphiphilic proteins involved in the formation of aerial structures such as spores or fruiting bodies. They form chemically robust layers which can only be dissolved in strong acids. These layers adhere to different surfaces, changing their wettability, and allow the binding of other proteins. Herein, the modification of diverse types of surfaces with Class I hydrophobins is reported, highlighting the applications of the coated surfaces. Indeed, these coatings can be exploited in several fields, spanning from biomedical to industrial applications, which include biosensing and textile manufacturing.

## 1. Introduction

In the last decade, several papers have reported that amyloids can fulfill important functional roles in a variety of biological processes of taxonomically distant organisms. Many organisms take advantage of the ability of polypeptides to form amyloids [1,2]. In filamentous fungi, amyloids are involved in numerous processes, e.g., in signal transduction mechanism, in which Nod-like receptors control the induction of programmed cell death [3]; controlling the translation termination by Sup35 [4] and the nitrogen catabolism by Ure2 [5]; and in the formation of aerial structures (spores or fruiting bodies) by amphipathic proteins known as hydrophobins (HFBs) [6]. HFBs self-assemble into an amphipathic membrane at hydrophilic : hydrophobic interfaces fulfilling many other fungal functions, such as the adherence of fungal structures to hydrophobic surfaces, including the surface of a host organism, thereby facilitating pathogenesis and playing a role in symbiosis [7]. Analysis of fungal genomes indicates that HFBs exist as gene families. Different HFBs are expressed at different stages in the fungal life cycle accomplishing specific functions [8].

The HFB family is composed of small proteins (<20 kDa) with high sequence variability, however they share a β-barrel motif in the 3D structure and a pattern of eight cysteine residues forming four disulfide bonds that stabilize the protein core [8]. These proteins are divided into two main classes based on their structural differences, such as the lengths of the inter-cysteine spaces, which determine their different properties. Class I HFBs assemble into insoluble polymeric layers composed of fibrillar structures known as rodlets and have a morphology like amyloid fibrils associated with diseases states [9,10,11]. These layers are extremely stable (resistant to treatment with hot 2% sodium dodecyl sulfate), can only be solubilized with harsh acid treatments (very concentrated formic acid or trifluoroacetic acid) and the soluble forms can polymerize back into rodlets under appropriate conditions [12]. Conversely, the layers formed by class II hydrophobins lack the fibrillary rodlet morphology and can be solubilized with organic solvents and detergents [13].

Only class I HFBs belong to the functional amyloid family, since the structural and morphological similarities between rodlets and amyloid fibrils have been confirmed many times. Indeed, rodlets bind amyloid-specific dyes (Congo Red, Thioflavin T) and their diffraction pattern displays typical reflections of amyloid structures (4.8 and 10–12 Å). [9].

The propensity of class I HFBs to self-assemble and the presence of disorder portions in their soluble forms, has precluded the achievement of crystals suitable for X-ray crystallography. However 3D structures of soluble class I HFBs have been obtained by nuclear magnetic resonance (NMR) studies for the EAS from *Neurospora crassa* [9], DewA from the fungus *Aspergillus nidulans* [14], MPG1 from the fungus *Magnaporthe oryzae* [15], and very recently for SC16 from *Schizophyllum commune* [16].

Both classes of HFBs have been exploited in many biotechnological applications [17]. Bioinspired coatings based on HFBs can offer novel opportunities for surface modification [13]. Over the last decade the surfaces coated with the layers of HFBs have been employed to immobilize several enzymes of industrial interest, and several materials coated with HFBs and their engineered variants have been proven effective for a wide range of biotechnological applications. Taking into consideration the topic of this special issue, this review is focused on the recent advances in the use of the functional amyloids class I HFBs in surface coating, organizing the reported results based on the typology of the surfaces.

## 2. Metal and Metalloid Functionalization

Thin films of titanium show unique chemical, optical, and electrical properties [18]. The interest on their fabrication is increasing due to their attractive applications (i.e., microelectronic devices, photonic materials, high-efficiency catalysts, environmental remediation, optical devices, and medical treatments). Santhiya et al. [19] set up a novel method for the aqueous phase deposition of smooth, nanocrystalline TiO_2_ thin films using a self-assembled HFB layer on a silicon substrate. In this report, the class I HFB used was H*Protein B, an engineered protein based on the class I HFB DewA from *A. nidulans*. A HFB layer on the silicon surface was prepared and used to deposit highly uniform nanocrystalline TiO_2_ thin films. Resistance and elasticity properties of the developed films were compatible with implant coatings and other biomedical devices.

Boeuf et al. [20] engineered DewA from *A. nidulans* by inserting the RGD sequence or the laminin globular domain (LG3 binding motif) at surface-accessible sites of the protein to functionalize the surfaces of orthopedic implants made of titanium. The purified proteins were used to produce surfaces that can enhance the adhesion of the human cells, while the adhesion of *Staphylococcus aureus* did not increase, thus minimizing the risk of bacterial infection.

Vmh2 from *Pleurotus ostreatus* was used to functionalize different surfaces, such as silicon, steel and gold. Vmh2 forms a chemically and mechanically stable layer of self-assembled proteins on crystalline silicon. This biomolecular film was exploited as a masking material, since the protein film perfectly protected the coated silicon surface during the standard KOH etching process [21]. The protein-modified silicon surface exhibited also an improvement in wettability and suitability for the immobilization of other proteins, such as BSA or the enzyme laccase, improving its stability [22].

**Figure 1:**
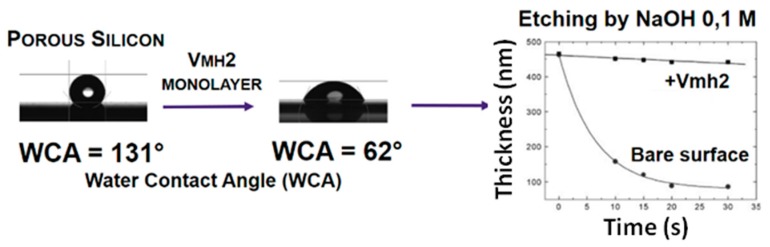
Change of wettability of PSi by Vmh2 and variation of PSi thickness with time, showing protection from etching by Vmh2.

The self-assembled Vmh2 layer also changed the wettability of the Porous silicon (PSi) structures and protected this nanocrystalline material from the basic dissolution process. PSi is a versatile material owing to its peculiar morphological, physical, and chemical properties [23] The major drawback of the “as etched” PSi is its chemical instability, since it is oxidized at room temperature by atmospheric oxygen [24]. The Vmh2 coating added chemical stability to PSi (Figure 1), without altering the sensing ability of this optical transducer, which can be a key tool for biomolecular experiments.

**Figure 2:**
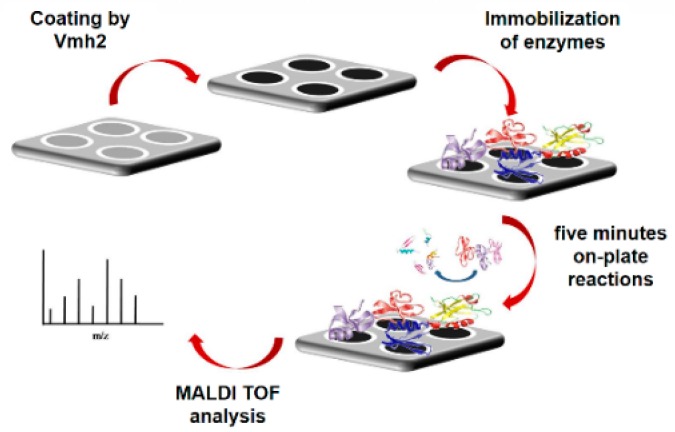
In situ reaction of trypsin immobilized on Vmh2 coated matrix-assisted laser desorption/ionization time-of-flight mass spectrometry (MALDI-TOF-MS) sample plate.

The sample-loading steel plate used in matrix-assisted laser desorption/ionization time-of-flight mass spectrometry (MALDI-TOF MS) was stably coated by the Vmh2 layer and the functionalized support can be reused, since Vmh2 can be de-polymerized and solubilized. The hybrid surface was able to homogenously adsorb peptides and proteins whereas salts or denaturants could be washed away with water, allowing fast and high-throughput on-plate desalting prior to MS analysis [25]. Moreover, the function of the Vmh2 coating was expanded by immobilizing enzymes of interest in proteomics (trypsin, V8 protease, PNGaseF, and alkaline phosphatase) on the steel surface. High sequence coverage of model proteins and analysis of a whole proteome (whey milk) were achieved by rapid and efficient multiple enzyme digestions, serially performed on plate (Figure 2) [26]. The same procedure provided the opportunity to discriminate blood provenance even when two different blood sources were present in a mixture [27]. Phosphatases or deglycosidase were also immobilized on-plate, allowing the study of proteins with post-translational modifications [26].

The spontaneous self-assembling of Vmh2 on the gold surface was verified by using the quartz crystal-microbalance (QCM) and confirmed by spectroscopic ellipsometry [28]. The Vmh2 layer stably assembled on the gold QCM electrode was also used to perform a quantitative analysis of the Vmh2-glucose interaction.

Additionally, gold nanoparticles (AuNPs) were synthesized, by a simple and novel process, in the presence of polyethylene-glycol (PEG), using Vmh2 to produce stable hybrid protein–metal nanoparticles, with outer surface rich in functional chemical groups [29]. Even though in the hybrid system the Vmh2 proteins were intrinsically bonded to the gold core, Vmh2-glucose interaction was confirmed, and the PEG-HFB-AuNPs was used in glucose monitoring [30].

## 3. Plastic Functionalization

Different plastic materials were modified using class I HFBs from both native and recombinant sources (Table 1). When heterologously produced, fused proteins composed of the HFB moiety and target proteins were successfully exploited. The modified materials were mainly used in the development of biomedical devices, examples of applications in biosensing and protein immobilization were demonstrated.

Platforms for protein immobilization were developed by using HGFI from *Grifola frondosa* native [31] and recombinant source [32]. On the other hand, the HFBs DewA and DewB from *A. nidulans* were fused to the laccase LccC from the same fungal source, developing an efficient system for laccase immobilization on polystyrene multiwell plates [33].

The exploitation of plastic surfaces coated with class I HFBs for biomedical devices was started by the pioneering work of Scholtmeijer et al. and Janssen and coworkers, who exploited the class I HFBs SC3 and SC4 (either native or recombinant engineered fused HFBs) in the functionalization of Teflon surfaces [34,35,36]. In that work, as well as in other examples, the main interest was in the development of biocompatible surfaces that allow both adhesion of human cells and tissue regeneration. Indeed, the HFBs were fused to different cell adhesion-mediating motifs and proved to be effective in the design of materials for regenerative medicine [20,39]. Moreover, Wang et al. and Zhao et al. [31,40] fused the HGFI to the vascular endothelial growth factor (VEGF), an effective molecule able to regulate the proliferation, migration, and survival pathways of endothelial cells [41].

Other examples of medical applications are the development of antibacterial devices [42] by the fusion of the bacteriocin pediocin PA-1 to HGFI. Furthermore, the layers formed by two fungal hydrophobins (Vmh2 and Pac3 from *A. sclerotigenum* [47]) reduce the biofilm formed by different strains of *Staphylococcus epidermidis* on polystyrene surfaces, without affecting the cell vitality [43]. Polymeric surfaces with enhanced lubricity and reduced surface friction were obtained using SC3 by Misra and coworkers [37]. Biliary plastic stents with delayed clogging process were developed thanks to the coating with the HFB, alone or in combination with heparin [38].

**Figure 3:**
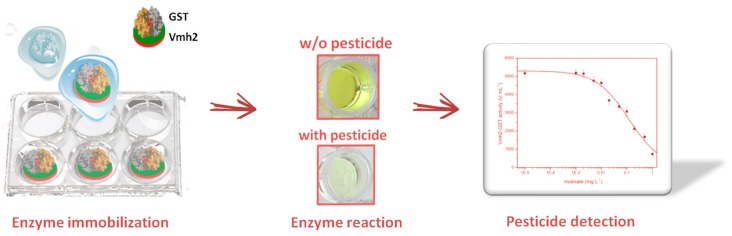
Pesticide biosensor developed on polystyrene multiwell plate coated with Vmh2-GST fused proteins

Considering biosensing applications, the EAS HFB from *N. crassa* fused to the yeast peptide pheromone α-factor was used in the detection and quantification of this pheromone [44]. Upon functionalization of polystyrene multiwell plate with a combination of HFBs either lacking or exposing the α-factor, an inverted enzyme-linked immunosorbent assay (ELISA) approach was developed yielding a novel kind of biosensor with the lowest limit of detection reported at the time of publication. Vmh2 from *P. ostreatus* fused to the enzyme glutathione-S-transferase (Vmh2-GST) was exploited for the quantification of the pesticides molinate and captan, acting as inhibitors of the enzymatic activity [45]. The fused protein efficiently functionalized the polystyrene multiwell plate for the development of high throughput analyses (Figure 3).

**Figure 4:**
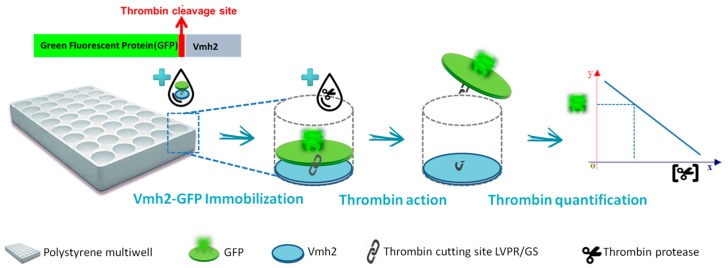
Thrombin biosensor developed on polystyrene multiwell plate coated with Vmh2-GFP fused proteins.

Vmh2 adhesion ability was also combined with the fluorescence emission of the Green Fluorescent Protein (GFP) by genetic fusion [46]. Vmh2-GFP was proven to be a smart and effective tool for the study of Vmh2 self-assembling and was used as the active biological element in the realization of an ultrasensitive thrombin biosensor. Since the two proteins were linked by the specific cutting site of the thrombin, a decrease in the fluorescence intensity of the sample was observed due to the cleavage of the linker by thrombin and the subsequent desorption of the GFP from the surface (Figure 4).

## 4. Carbon Nanotubes and 2D Materials Functionalization

**Figure 5:**
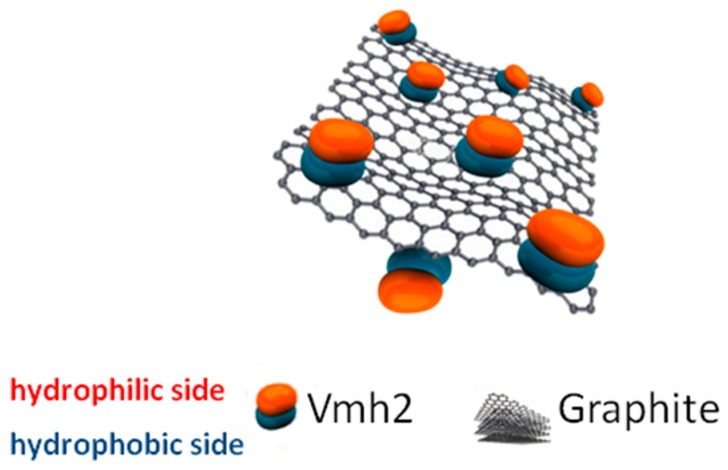
Biofunctionalized graphene produced by in situ exfoliation of graphite in the presence of Vmh2.

Since 2D materials possess high surface area to volume ratio, they can be exploited in enzyme immobilization, obtaining high enzyme loading and increasing the reaction kinetics, thus improving biocatalytic efficiency for industrial applications. Carbon nanotubes (CNT), graphene, and the semiconducting transition metal dichalcogenides MoS_2_ and WS_2_ were dispersed and coated by class I HFBs [48,49,50,51]. Few layer microsheets of graphene were produced and dispersed by ultrasonic wave exfoliation of low-cost graphite in the presence of Vmh2 in water-ethanol solutions (Figure 5) [49]. Notably, the non-covalent nature of the amphiphilic protein–carbon interactions preserved the band structure of sp^2^-carbon lattice. The functionalized bio-hybrid material was endowed with the self-assembling properties of Vmh2 (including the ability to form homogeneous films), controlled by environmental factors, and is a valuable material for biotechnological applications, such as sensing, nanomedicine, and bioelectronic technologies.

When Vmh2 was interfaced to MoS_2_ and WS_2_ nanosheets their ζ-potential could be tuned to control the stability of the dispersions. Stable liquid dispersions of high quality few-layered, photoluminescent and bio-functionalized nanosheets of 2D materials were produced [50].

## 5. Functionalization of Other Materials

### 5.1. Biosensing and Biomedical Applications

The recombinant HFB of the fungus *Pisolithus tinctorius* HYDPt-1 was used to immobilize small, electroactive molecules on three different electrode substrates: glassy carbon electrode (GCE), thin mercury film electrode (TMFE) and hydrophilic surfaces such as a gold electrode (GE) [52]. These promising results were evolved in the settings of stable, enzyme-based catalytic surfaces for applications in biosensing [53]. Two redox enzymes, glucose oxidase and horseradish peroxidase, which are utilized in biosensing, were immobilized on SC3 HFB coated GCE and were active for more than one month.

The applications of HGFI in surface wettability conversion on mica (patterning applications), glass (cell culture and protein fixation) and polydimethylsiloxane (biomedical devices), were investigated by Hou et al. [54]. The coated surfaces were used as platform for antibody immobilization to set up an immunoassay system, thus showing a feasible strategy for biosensor device fabrication.

The ability of class I HFB to functionalize glass surface was demonstrated by Rieder and coworkers [55]. Recently, the spontaneous self-assembling of Vmh2 at liquid–solid interfaces was exploited to achieve the highly homogeneous glass functionalization in 4 min, coating 1880 cm^2^ of glass per mg of protein [56]. The Vmh2-coated glass slides were proven to immobilize not only proteins, but also nanomaterials such as graphene oxide (GO) and cadmium telluride (CdTe) quantum dots (QDs). This novel glass substrate can be amenable for optical biosensing applications in the microarray format. Moreover, two HFB proteins were exploited in dental repair applications. DewA_4 and DewA_5 showed binding abilities to hydroxyapatite in a mouthwash formulation and an increased nucleation in artificial saliva [57].

### 5.2. Textiles Finishing Processes

The non-toxic and biodegradable properties of HFBs can have great potential in textile surface modification, changing properties such as wettability, and flame resistance. After HFB deposition, hydrophobic fabrics resulted in a significant hydrophilization, while hydrophilic textiles attained strong hydrophobic character [58]. Cotton fabrics coated by the H*Protein B HFB displayed an enhanced flame resistance, suggesting that the use of these proteins can be an alternative strategy for the design of sustainable and green flame retardants [59]. The normal finishing agents that confer the anti-microbial resistance to textiles have low adherence and poor uniformity. The use of H*Protein A and H*Protein B avoided these drawbacks and the formed uniform layer of HFBs and antimicrobial agents (Ag and ZnO) on cotton/poly ester fabrics inhibited different bacterial species [60].

### 5.3. Biocatalytic Transformations

Palomo et al. [61] used *P. ostreatus* HFBs to build a functionalized agarose support to immobilize different lipases. The immobilized lipases underwent the typical mechanism of interfacial activation on hydrophobic supports. Hence the time required for the immobilization procedure was offset by the improvement of the catalytic activity, stability and enantioselectivity of the enzyme.

More recently, the physiological role of HFBs to cover the wall of mycelium was used to change the surface characteristics of *Pichia pastoris* cells, opening the frontiers to the development of new high efficiency cell catalysts [62]. Surface display of fungal HFBs SC3 successfully modified the hydrophobicity of the surface of yeast cells.

## 6. Conclusion

HFBs can represent robust and reliable green alternatives to chemical strategies in surface functionalization. Albeit the number of applications of Class I HFBs is quite large in laboratory scale, the full potential of these proteins is yet to be realized. The main bottleneck for their utilization is the lack of production systems at industrial level. Most HFBs cannot yet be produced in gram per liter quantities, thus limiting their use. Currently, only BASF succeeded in the production of the two recombinant class I HFBs, H*Protein A and B, in quantities sufficient for large-scale applications [63]. This result suggests that industrial applications of hydrophobins are expected in the near future.

## Figures and Tables

**Table 1:** Class I hydrophobins used in functionalization of plastic materials.

Application	HFB	Source	Surface	Refs.
Medical	eng SC3 ^1^	*S. commune*	Teflon	[34,35,36]
	SC3—RGD ^2^			
	eng SC3—RGD ^1,2^			
	eng SC3 ^1^	*S. commune*		[34,35,36]
	SC3—RGD ^2^			
	SC3	*S. commune*		[34,35,36]
	SC4			
	SC3	*S. commune*	Polystyrene	[37]
			Copolymer of benzoyl-1,4 phenylene and 1,3-phenylene	
	eng DewA ^1^	*A. nidulans*	Plastic biliary stent	[38]
	DewA	*A. nidulans*	Polystyrene	[20]
	DewA–RGD ^2^			
	DewA–LG3 ^2^			
	HGFI—TPS ^2^	*G. frondosa*	Polycaprolattone	[39]
	HGFI—VGF ^2^	*G. frondosa*		[40]
	HGFI—VGF ^2^	*G. frondosa*		[41]
	HGFI—PA1 ^2^	*G. frondosa*		[42]
	Vmh2	*P. ostreatus*	Polystyrene	[43]
	Pac3	*Acremonium sclerotigenum*		
Biosensing	EAS	*N. crassa*	Polystyrene	[44]
	EAS-α ^2^			
	Vmh2-GST ^2^	*P. ostreatus*		[45]
	Vmh2-GFP ^2^	*P. ostreatus*		[46]
Immobilization	HGFI	*G. frondosa*	Polystyrene	[31]
	rHGFI ^3^	*G. frondosa*		[32]
	DewA–LccC ^2^	*A. nidulans*		[33]
	DewB–LccC ^2^			

^1^ Recombinant engineered protein; ^2^ Recombinant fused protein; ^3^ Recombinant protein.
